# The human brain: The final frontier and the wild west

**DOI:** 10.1172/JCI173352

**Published:** 2023-07-17

**Authors:** Salman E. Qasim

**Affiliations:** Department of Psychiatry, Icahn School of Medicine at Mount Sinai, New York, New York, USA.

“I can do it, I promise!”

I watched as the patient sweated her way through the third experiment I had asked her to do that day. She had consented to be a part of our research project and was determined to contribute — she had told me that it wasn’t every day that a person had electrodes implanted in their brain, and what else was she doing while she waited to have seizures that the epileptologists could track? I was a Ph.D. student at the time, conducting this research by the book. And the neural data coming through my monitor was beautiful — neuronal action potentials and brain waves recorded directly from the amygdala, the frontal cortex, and the hippocampus. This human brain data provided a rare window into the specific neural mechanisms that construct our feelings, our choices, and our memories. Data like this could lead to new therapies for a dizzying array of neurological and psychiatric disorders — the goal I’ve worked towards for the last decade.

So why was I feeling bad?

Recording from a neurosurgical patient’s brain while they perform cognitive experiments provides an incredibly rare opportunity to directly test theories of how brain activity constructs complex cognitive functions that are hard to study in animals. Such research participation is carefully monitored and governed by Institutional Review Boards (IRBs). Numerous studies have outlined how such research poses very minimal risk to patients (1). Numerous clinicians and researchers have provided practical guides to carrying out such research in an ethical, patient-first manner (2, 3). Clinician and nurse oversight is constant, and the patient is told repeatedly that they may withdraw their consent at any point with no detriment to their clinical treatment.

And still, watching this patient earnestly struggle to pay attention to the experiment instructions, I felt bad.

“Bad” is, of course, not a scientific feeling, nor does it portend legal liability issues. And all the research I had done was strictly within the limits defined by the IRB and scientific research norms. But that sticky feeling predates scientific and legal norms (by a lot!) and I have always considered it worth my attention, so I asked myself: am I paying enough attention to the lived experience of the person actually participating in the research?

I take comfort in standing behind the IRB’s blanket declaration that I am allowed to conduct this research with consenting patients, but as a scientist I should know better than to mindlessly apply a blanket declaration to an individual observation. Ultimately, the IRB wasn’t in the room with this specific patient, in this specific moment — I was. Had I considered the possibility that this specific patient might have people-pleasing or self-sacrificial tendencies? Had I paid enough attention to her nonverbal cues? Simply put, had I, the experimenter, talked to her enough?

I asked the patient why she had agreed to participate in the research, beyond “not having anything else to do”. She thought about it for a moment. “I feel terrible. My jaws are constantly in pain because this headwrap is so tight, and I wish I could just stand up and walk to the bathroom. I can’t wait to get out of here. While I’m here, I might as well try to benefit at least one other person in the world with whatever you find out from my brain.” I was touched by her desire to transform her pain into someone else’s benefit. I also understood that it might not be my place to undermine her agency by preventing her from participating in more research. But I was also mindful of the possibility that the potential benefits of the research to others could have acted almost as a coercive influence. I didn’t leave her room with any easy or obvious answers about the ideal ethical approach.

Luckily, my burgeoning thought process has been met with a similar emphasis in the neuroscientific zeitgeist. Shortly after this experience, Columbia University, where I completed my Ph.D., invited philosophers and ethicists to discuss Neuroethics as a part of the Neuroethics Workgroup of the NIH BRAIN Initiative, evidence for a broad (funded!) shift towards thoughtful human neuroscience research. This has even inspired many major intracranial research projects to include subcontracts for bioethicists. As intracranial human neuroscience expands in parallel with my own scientific career I hope to help shape the conversation around ethical research practices, especially as for-profit companies, such as Neuralink, emerge at the forefront of such research.

As I left that patient’s room, I may not have had all the answers. But I did leave with a sense of responsibility to approach intracranial human neuroscience research on a more personal basis, a desire to continue to do better, and a belief that for the time being, this was enough.

## Version 1 07/17/2023

Electronic publication
